# (*E*)-*N*′-(4-Methoxy­benzyl­idene)benzohydrazide

**DOI:** 10.1107/S1600536809049988

**Published:** 2009-11-25

**Authors:** Jian-Xia Gou, Ming-Zhi Song, Chuan-Gang Fan, Zhong-Nian Yang

**Affiliations:** a College of Chemistry and Chemical Technology, Binzhou University, Binzhou 256600, Shandong, People’s Republic of China; b College of Chemistry and Chemical Engineering, China University of Petroleum, Qingdao, Shandong 266555, People’s Republic of China

## Abstract

In the title mol­ecule, C_15_H_14_N_2_O_2_, the dihedral angle between the benzene rings is 5.93 (17)°. In the crystal, inter­molecular N—H⋯O hydrogen bonds link the mol­ecules into chains propagating in [010].

## Related literature

For properties of Schiff base ligands, see: Cozzi *et al.* (2004 ▶). For related crystal structures, see: Fun *et al.* (2008 ▶); Cui *et al.* (2009 ▶); Nie (2008 ▶).
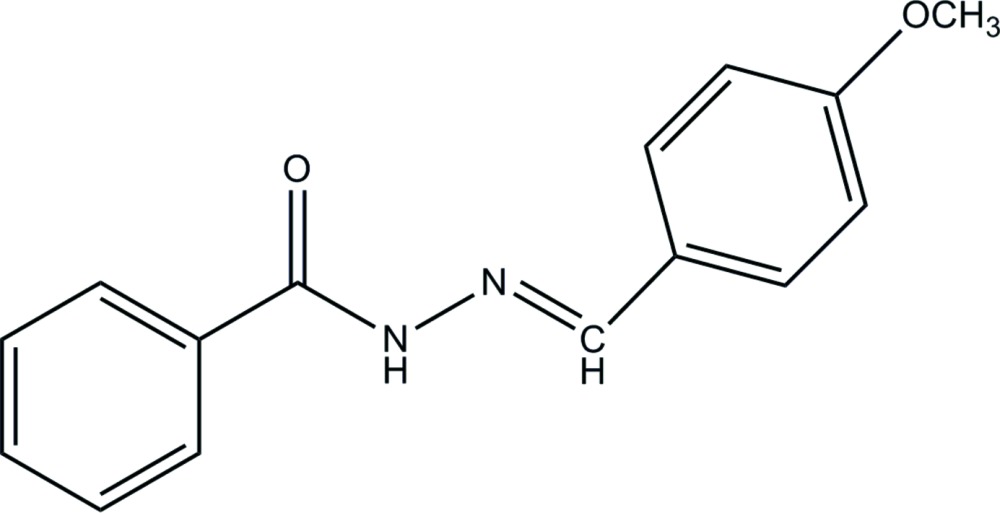


## Experimental

### 

#### Crystal data

C_15_H_14_N_2_O_2_
*M* *_r_* = 254.28
Orthorhombic, 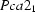

*a* = 31.414 (3) Å
*b* = 5.1067 (5) Å
*c* = 8.1336 (9) Å
*V* = 1304.8 (2) Å^3^
*Z* = 4
Mo *K*α radiation
μ = 0.09 mm^−1^
*T* = 298 K
0.49 × 0.48 × 0.30 mm

#### Data collection

Bruker SMART APEX CCD area-detector diffractometer
Absorption correction: multi-scan (*SADABS*; Sheldrick, 1996 ▶) *T*_min_ = 0.958, *T*_max_ = 0.974
2220 measured reflections
1239 independent reflections
920 reflections with *I* > 2σ(*I*)
*R*_int_ = 0.037

#### Refinement

*R*[*F* ^2^ > 2σ(*F* ^2^)] = 0.042
*wR*(*F* ^2^) = 0.106
*S* = 1.03
1239 reflections
173 parameters
1 restraint
H-atom parameters constrained
Δρ_max_ = 0.18 e Å^−3^
Δρ_min_ = −0.14 e Å^−3^

### 

Data collection: *SMART* (Siemens, 1996 ▶); cell refinement: *SAINT* (Siemens, 1996 ▶); data reduction: *SAINT*; program(s) used to solve structure: *SHELXS97* (Sheldrick, 2008 ▶); program(s) used to refine structure: *SHELXL97* (Sheldrick, 2008 ▶); molecular graphics: *SHELXTL* (Sheldrick, 2008 ▶); software used to prepare material for publication: *SHELXTL*.

## Supplementary Material

Crystal structure: contains datablocks I, global. DOI: 10.1107/S1600536809049988/bq2172sup1.cif

Structure factors: contains datablocks I. DOI: 10.1107/S1600536809049988/bq2172Isup2.hkl

Additional supplementary materials:  crystallographic information; 3D view; checkCIF report

## Figures and Tables

**Table 1:** Hydrogen-bond geometry (Å, °)

*D*—H⋯*A*	*D*—H	H⋯*A*	*D*⋯*A*	*D*—H⋯*A*
N1—H1⋯O1^i^	0.86	2.17	2.961 (2)	152

Symmetry code: (i) 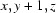.

**Fig. 1.**
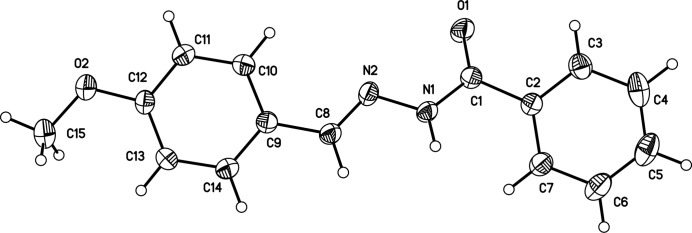
A view of (I) showing the atomic numbering scheme and 30% probability displacement ellipsoids.

**Fig. 2.**
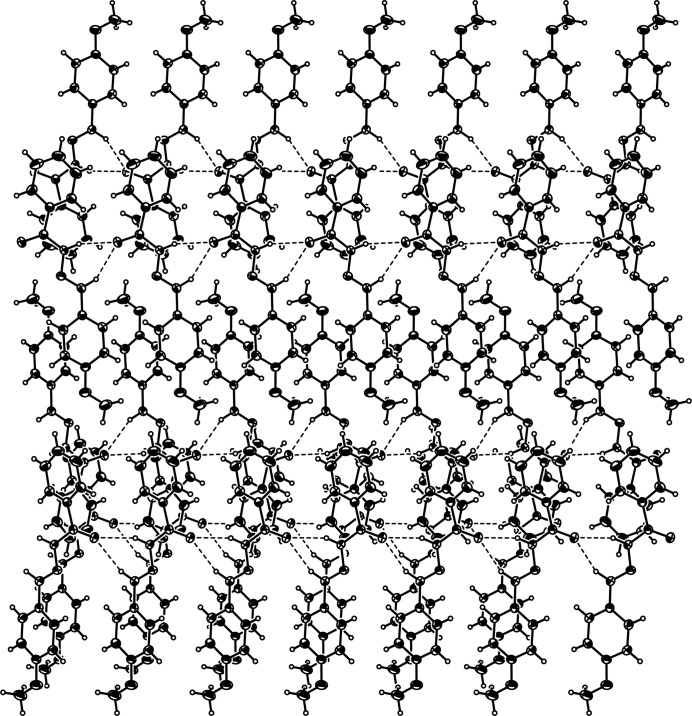
The packing of (I) builted from the connections of weak intermolecular N—H···O hydrogen bonds with dashed lines.

## supplementary crystallographic information

### Comment

Schiff bases are popular ligands in coordination chemistry due to their ease of synthesis and their ability to be readily modified both electronically and sterically. Mixed-donor Schiff bases have been used extensively in catalysis (Cozzi, 2004).

In (I), (Fig. 1), the bond lengths an angles are normal and are comparable to the values observed in similar compounds (Nie *et al.*, 2008; Fun *et al.*, 2008; Cui *et al.*, 2009).

In the crystal structure, the C8=N2 bond length in the molecule is 1.269 (3) °, showing the double-bond character. Meanwhile, the dihedral angle between the benzene ring (C2-C7) and the benzene ring (C9-C14) in the Schiff base molecule is 5.93 (17) °, indicating that the two aromatic ring planes are almost coplanar. Moreover, the crystal supramolecular structure was built from the connections of weak intermolecular N—H···O hydrogen bonds, as shown in table 1, and these hydrogen bonds link molecules into one-dimensional chains propagated in direction [010].

### Experimental

Benzohydrazide (5.0 mmol), 20 ml ethanol and 4-methoxybenzaldehyde (5.0 mmol) were mixed in 50 ml flash. After refluxing 3 h, the resulting mixture was cooled to room temperature, and recrystalized from ethanol, and afforded the title compound as a crystalline solid. Elemental analysis: calculated for C_15_H_14_N_2_O_2_: C 70.85, H 5.55, N 11.02%; found: C 70.78, H 5.64, N 11.13%.

### Refinement

All H atoms were placed in geometrically idealized positions (N—H=0.86 and C—H=0.93–0.96 Å) and treated as riding on their parent atoms, with *U*_iso_(H) = 1.2–1.5*U*_eq_(C) (C,*N*). Because of the meaningless of the absolute structure parameter, 981 Friedel-pairs were merged before final refinement.

### Crystal data

**Table:**

C_15_H_14_N_2_O_2_	*F*(000) = 536
*M**_r_* = 254.28	*D*_x_ = 1.294 Mg m^−^^3^
Orthorhombic, *P**c**a*2_1_	Mo *K*α radiation, λ = 0.71073 Å
Hall symbol: P 2c -2ac	Cell parameters from 1475 reflections
*a* = 31.414 (3) Å	θ = 2.6–22.4°
*b* = 5.1067 (5) Å	µ = 0.09 mm^−^^1^
*c* = 8.1336 (9) Å	*T* = 298 K
*V* = 1304.8 (2) Å^3^	Block, colourless
*Z* = 4	0.49 × 0.48 × 0.30 mm

### Data collection

**Table:**

Bruker SMART APEX CCD area-detector diffractometer	1239 independent reflections
Radiation source: fine-focus sealed tube	920 reflections with *I* > 2σ(*I*)
graphite	*R*_int_ = 0.037
phi and ω scans	θ_max_ = 25.0°, θ_min_ = 1.3°
Absorption correction: multi-scan (*SADABS*; Sheldrick, 1996)	*h* = 0→36
*T*_min_ = 0.958, *T*_max_ = 0.974	*k* = 0→6
2220 measured reflections	*l* = −9→9

### Refinement

**Table:**

Refinement on *F*^2^	Primary atom site location: structure-invariant direct methods
Least-squares matrix: full	Secondary atom site location: difference Fourier map
*R*[*F*^2^ > 2σ(*F*^2^)] = 0.042	Hydrogen site location: inferred from neighbouring sites
*wR*(*F*^2^) = 0.106	H-atom parameters constrained
*S* = 1.03	*w* = 1/[σ^2^(*F*_o_^2^) + (0.051*P*)^2^ + 0.0327*P*] where *P* = (*F*_o_^2^ + 2*F*_c_^2^)/3
1239 reflections	(Δ/σ)_max_ < 0.001
173 parameters	Δρ_max_ = 0.18 e Å^−^^3^
1 restraint	Δρ_min_ = −0.14 e Å^−^^3^

### Special details

**Table:**

Geometry. All e.s.d.'s (except the e.s.d. in the dihedral angle between two l.s. planes) are estimated using the full covariance matrix. The cell e.s.d.'s are taken into account individually in the estimation of e.s.d.'s in distances, angles and torsion angles; correlations between e.s.d.'s in cell parameters are only used when they are defined by crystal symmetry. An approximate (isotropic) treatment of cell e.s.d.'s is used for estimating e.s.d.'s involving l.s. planes.
Refinement. Refinement of *F*^2^ against ALL reflections. The weighted *R*-factor *wR* and goodness of fit *S* are based on *F*^2^, conventional *R*-factors *R* are based on *F*, with *F* set to zero for negative *F*^2^. The threshold expression of *F*^2^ > σ(*F*^2^) is used only for calculating *R*-factors(gt) *etc*. and is not relevant to the choice of reflections for refinement. *R*-factors based on *F*^2^ are statistically about twice as large as those based on *F*, and *R*- factors based on ALL data will be even larger.

### Fractional atomic coordinates and isotropic or equivalent isotropic displacement parameters (Å^2^)

**Table:**

	*x*	*y*	*z*	*U*_iso_*/*U*_eq_	
N1	0.32900 (6)	0.0513 (4)	0.8803 (3)	0.0446 (6)	
H1	0.3182	0.2051	0.8691	0.053*	
N2	0.37282 (6)	0.0199 (4)	0.8715 (3)	0.0448 (6)	
O1	0.31604 (5)	−0.3787 (3)	0.9244 (3)	0.0612 (6)	
O2	0.57114 (5)	0.2572 (4)	0.8020 (3)	0.0618 (6)	
C1	0.30295 (7)	−0.1548 (5)	0.9062 (4)	0.0414 (6)	
C2	0.25645 (8)	−0.0907 (4)	0.9049 (4)	0.0404 (6)	
C3	0.22924 (9)	−0.2535 (6)	0.9909 (4)	0.0522 (8)	
H3	0.2400	−0.3961	1.0487	0.063*	
C4	0.18566 (10)	−0.2029 (7)	0.9905 (5)	0.0688 (11)	
H4	0.1673	−0.3098	1.0501	0.083*	
C5	0.16978 (9)	0.0027 (7)	0.9031 (6)	0.0707 (10)	
H5	0.1407	0.0366	0.9043	0.085*	
C6	0.19643 (9)	0.1599 (6)	0.8133 (5)	0.0653 (10)	
H6	0.1853	0.2959	0.7505	0.078*	
C7	0.23964 (8)	0.1159 (5)	0.8162 (4)	0.0506 (8)	
H7	0.2577	0.2262	0.7579	0.061*	
C8	0.39287 (8)	0.2202 (5)	0.8213 (4)	0.0437 (7)	
H8	0.3775	0.3676	0.7898	0.052*	
C9	0.43914 (7)	0.2271 (5)	0.8114 (4)	0.0399 (6)	
C10	0.46450 (8)	0.0478 (5)	0.8944 (4)	0.0478 (7)	
H10	0.4517	−0.0854	0.9547	0.057*	
C11	0.50810 (8)	0.0635 (5)	0.8889 (4)	0.0479 (7)	
H11	0.5245	−0.0577	0.9460	0.058*	
C12	0.52766 (7)	0.2591 (5)	0.7988 (4)	0.0420 (6)	
C13	0.50332 (9)	0.4391 (5)	0.7160 (4)	0.0472 (7)	
H13	0.5163	0.5708	0.6550	0.057*	
C14	0.45933 (8)	0.4235 (5)	0.7239 (4)	0.0485 (8)	
H14	0.4430	0.5479	0.6691	0.058*	
C15	0.59304 (9)	0.4528 (7)	0.7113 (5)	0.0735 (10)	
H15A	0.5848	0.4435	0.5978	0.110*	
H15B	0.6232	0.4249	0.7203	0.110*	
H15C	0.5860	0.6224	0.7544	0.110*	

### Atomic displacement parameters (Å^2^)

**Table:**

	*U*^11^	*U*^22^	*U*^33^	*U*^12^	*U*^13^	*U*^23^
N1	0.0362 (12)	0.0317 (11)	0.0658 (16)	0.0055 (9)	−0.0014 (13)	0.0011 (12)
N2	0.0331 (12)	0.0412 (12)	0.0600 (16)	0.0020 (10)	−0.0012 (13)	−0.0067 (12)
O1	0.0481 (10)	0.0330 (10)	0.1026 (18)	0.0060 (8)	−0.0035 (13)	0.0002 (12)
O2	0.0379 (11)	0.0707 (13)	0.0766 (14)	−0.0043 (10)	−0.0008 (12)	0.0158 (12)
C1	0.0410 (13)	0.0339 (13)	0.0492 (17)	−0.0011 (12)	−0.0008 (16)	−0.0051 (15)
C2	0.0402 (14)	0.0369 (13)	0.0439 (17)	−0.0002 (11)	−0.0016 (15)	−0.0060 (16)
C3	0.0510 (18)	0.0496 (17)	0.056 (2)	−0.0085 (14)	0.0014 (16)	−0.0045 (16)
C4	0.050 (2)	0.082 (3)	0.075 (3)	−0.0216 (18)	0.0150 (19)	−0.018 (2)
C5	0.0399 (16)	0.079 (2)	0.093 (3)	0.0042 (16)	−0.011 (2)	−0.022 (3)
C6	0.0501 (19)	0.060 (2)	0.086 (3)	0.0124 (16)	−0.0138 (19)	−0.009 (2)
C7	0.0453 (17)	0.0448 (15)	0.062 (2)	0.0023 (13)	−0.0056 (16)	−0.0029 (17)
C8	0.0398 (14)	0.0357 (14)	0.0556 (19)	0.0035 (12)	−0.0036 (14)	0.0009 (14)
C9	0.0398 (14)	0.0342 (14)	0.0456 (16)	0.0011 (12)	0.0002 (15)	−0.0049 (14)
C10	0.0451 (15)	0.0416 (14)	0.0566 (19)	0.0012 (12)	0.0025 (17)	0.0087 (18)
C11	0.0428 (15)	0.0446 (15)	0.0563 (19)	0.0049 (12)	−0.0057 (17)	0.0068 (18)
C12	0.0336 (15)	0.0442 (15)	0.0482 (16)	−0.0017 (13)	0.0013 (15)	−0.0061 (14)
C13	0.0444 (17)	0.0428 (17)	0.0542 (19)	−0.0055 (13)	0.0028 (16)	0.0076 (15)
C14	0.0444 (18)	0.0408 (16)	0.060 (2)	0.0063 (13)	−0.0031 (16)	0.0051 (15)
C15	0.0471 (19)	0.081 (2)	0.092 (3)	−0.0165 (16)	−0.0041 (18)	0.013 (2)

### Geometric parameters (Å, °)

**Table:**

N1—C1	1.350 (3)	C6—H6	0.9300
N1—N2	1.388 (2)	C7—H7	0.9300
N1—H1	0.8600	C8—C9	1.456 (3)
N2—C8	1.269 (3)	C8—H8	0.9300
O1—C1	1.224 (3)	C9—C14	1.384 (4)
O2—C12	1.366 (3)	C9—C10	1.389 (4)
O2—C15	1.420 (4)	C10—C11	1.373 (3)
C1—C2	1.497 (3)	C10—H10	0.9300
C2—C3	1.382 (4)	C11—C12	1.383 (4)
C2—C7	1.383 (3)	C11—H11	0.9300
C3—C4	1.393 (4)	C12—C13	1.372 (4)
C3—H3	0.9300	C13—C14	1.386 (4)
C4—C5	1.363 (5)	C13—H13	0.9300
C4—H4	0.9300	C14—H14	0.9300
C5—C6	1.370 (5)	C15—H15A	0.9600
C5—H5	0.9300	C15—H15B	0.9600
C6—C7	1.376 (4)	C15—H15C	0.9600
			
C1—N1—N2	121.29 (19)	N2—C8—H8	118.9
C1—N1—H1	119.4	C9—C8—H8	118.9
N2—N1—H1	119.4	C14—C9—C10	117.7 (2)
C8—N2—N1	114.6 (2)	C14—C9—C8	120.2 (2)
C12—O2—C15	118.0 (2)	C10—C9—C8	122.0 (3)
O1—C1—N1	122.9 (2)	C11—C10—C9	121.2 (3)
O1—C1—C2	122.2 (2)	C11—C10—H10	119.4
N1—C1—C2	114.8 (2)	C9—C10—H10	119.4
C3—C2—C7	119.1 (2)	C10—C11—C12	120.2 (3)
C3—C2—C1	117.9 (2)	C10—C11—H11	119.9
C7—C2—C1	122.9 (2)	C12—C11—H11	119.9
C2—C3—C4	119.7 (3)	O2—C12—C13	124.8 (3)
C2—C3—H3	120.2	O2—C12—C11	115.4 (2)
C4—C3—H3	120.2	C13—C12—C11	119.8 (2)
C5—C4—C3	120.3 (3)	C12—C13—C14	119.7 (3)
C5—C4—H4	119.9	C12—C13—H13	120.2
C3—C4—H4	119.9	C14—C13—H13	120.2
C4—C5—C6	120.4 (3)	C9—C14—C13	121.5 (3)
C4—C5—H5	119.8	C9—C14—H14	119.2
C6—C5—H5	119.8	C13—C14—H14	119.2
C5—C6—C7	119.9 (3)	O2—C15—H15A	109.5
C5—C6—H6	120.1	O2—C15—H15B	109.5
C7—C6—H6	120.1	H15A—C15—H15B	109.5
C6—C7—C2	120.7 (3)	O2—C15—H15C	109.5
C6—C7—H7	119.7	H15A—C15—H15C	109.5
C2—C7—H7	119.7	H15B—C15—H15C	109.5
N2—C8—C9	122.2 (2)		

### Hydrogen-bond geometry (Å, °)

**Table:**

*D*—H···*A*	*D*—H	H···*A*	*D*···*A*	*D*—H···*A*
N1—H1···O1^i^	0.86	2.17	2.961 (2)	152

Symmetry codes: (i) *x*, *y*+1, *z*.
